# Lysine 268 adjacent to transmembrane helix 5 of hamster P‐glycoprotein is the major photobinding site of iodomycin in CHO B30 cells

**DOI:** 10.1002/2211-5463.13112

**Published:** 2021-02-28

**Authors:** Annette Demmer, Hubert Thole, Manfred Raida, Burkhard Tümmler

**Affiliations:** ^1^ Klinische Forschergruppe Klinik für Pädiatrische Pneumologie Allergologie und Neonatologie Medizinische Hochschule Hannover Germany; ^2^ Singapore Lipidomics Incubator (SLING) Life Sciences Institute National University of Singapore Singapore; ^3^ Biomedical Research in Endstage and Obstructive Lung Disease Hannover Deutsches Zentrum für Lungenforschung (DZL) Medizinische Hochschule Hannover Germany

**Keywords:** ABC transporter, anthracycline, drug‐binding site, Edman sequencing, multidrug resistance, P‐glycoprotein

## Abstract

P‐glycoprotein (Pgp) detoxifies cells by exporting hundreds of chemically dissimilar hydrophobic and amphipathic compounds and is implicated in multidrug resistance (MDR) in the treatment of cancers. Photoaffinity labeling of plasma membrane vesicles of MDR CHO B30 cells with the anthracycline [^125^I]‐iodomycin, subsequent sequential cleavage with BNPS‐skatol and endoproteinase Lys‐C, and the Edman sequencing of the purified photoaffinity‐labeled peptide identified the lysine residue at position 268 in the hamster Pgp primary sequence as the major photobinding site of iodomycin in CHO B30 cells. Lysine 268 is located adjacent to the cytosolic terminus of transmembrane 5. According to thermodynamic and kinetic analyses, this location should present the equilibrium binding site of ATP‐free Pgp for daunomycin and iodomycin in B30 cells.

## Graphical abstract

The multidrug transporter P‐glycoprotein (Pgp) exports hundreds of chemically dissimilar compounds. Whilst crystallography and cryo‐electron microscopy localized drug binding sites of reconstituted recombinant Pgp in the centre or the ectoplasmic site of the bilayer, we identified the binding site for the anthracycline iodomycin in native plasma membranes at the border between the transmembrane domains and the nucleotide binding sites of Pgp.

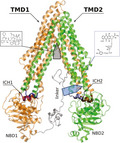

**Abbreviations**
ABCB1: ATP‐binding cassette subfamily B member 1
CHO: Chinese hamster ovary
Cryo‐EM: cryo‐electron microscopy
HPLC: high‐performance liquid chromatography
IF: inward‐facing conformation
Lys‐C: *Achromobacter lyticus* lysyl endopeptidase C
MDR: multidrug resistance
NBD: nucleotide‐binding domain
OF: outward‐facing conformation
Pgp: P‐glycoprotein
PTH: phenylthiohydantoin
TFA: trifluoroacetic acid
TMD: transmembrane domain
TMH: transmembrane helix

The ATP‐binding cassette subfamily B member 1 (ABCB1, UniProt No. P08183) multidrug transporter P‐glycoprotein (Pgp) is involved in the clearance of xenobiotics in mammals and is implicated in cancer resistance to chemotherapy [1, 2]. Pgp detoxifies cells by exporting hundreds of chemically dissimilar hydrophobic and amphipathic compounds. This substrate promiscuity is a hallmark of Pgp activity. In its inward‐facing conformation (IF), the two cytosolic nucleotide‐binding domains (NBDs) are separated from each other and the transmembrane domains (TMDs) form a large internal drug‐binding cavity that is accessible from the cytoplasm and the inner leaflet of the plasma membrane for drug entry [3]. In the outward‐facing conformation (OF), the two NBDs make extensive contacts and the drug‐binding cavity is reoriented to the extracellular space and compressed to preclude drug binding [4, 5]. The IF‐to‐OF transition requires ATP turnover, which is accelerated by substrate binding [6, 7].

This current model of the substrate‐coupled conformational cycle of Pgp has mainly been derived from recent studies on recombinant Pgp in model membranes by X‐ray crystallography [3, 5], cryo‐electron microscopy (Cryo‐EM) [4, 8], and double electron–electron resonance spectroscopy of spin‐label pairs in positions that fingerprint the IF‐to‐OF transition [6]. Back in 1976, Juliano and Ling discovered Pgp in colchicine‐resistant cells by labeling cell surface carbohydrates [9]. To get a handle on the biochemical features of the highly hydrophobic Pgp, mutant lines of Chinese hamster ovary (CHO) cells were selected that overexpress Pgp due to the exposure to a cytotoxic agent like daunomycin or colchicine [10]. These CHO lines could be exploited for the first biochemical visualization of Pgp in gels [11] and immunoblot [12, 13]. To study drug binding of Pgp in its native plasma membrane environment, we chose the multidrug‐resistant (MDR) CHO B30 cell line to examine the high‐affinity binding of iodomycin [14], the Bolton–Hunter derivative of the anthracycline daunomycin, to Pgp in plasma membranes by equilibrium, kinetic, and photoaffinity labeling studies [15]. The covalent photobinding site to [^125^I]‐iodomycin was localized to the amino acid sequences 230–312 of hamster Pgp [16]. Here, we report on the identification of lysine 268 Pgp as the major photobinding site of iodomycin in CHO B30 cells.

## Materials and methods

### Cell culture and Preparation of plasma membrane vesicles

CHO B30 cells were grown in alpha‐minimum essential medium supplemented with glutamine, nucleosides, 10% calf serum, and 30 µg·mL^−1^ colchicine [13]. Plasma membrane vesicles were prepared from 50 culture plates per batch [16].

### Detection of radioactivity

For the detection of radioactivity, the gels were exposed to a Fuji imaging plate type BAS‐IIIs. Liquid samples were measured in a Compu γ‐counter (LKB; Wallac, Vienna, Austria).

### Photoaffinity labeling

[^125^I]‐Iodomycin (2000 Ci·mmol^−1^) was prepared from daunomycin by reaction with [^125^I]‐labeled Bolton–Hunter reagent as described previously [15]. For photoaffinity labeling, 75 µg plasma membrane protein and [^125^I]‐labeled iodomycin (0.10 pmol per 10 µg protein) were suspended in 150 µL 5 mm Tris/HCl, pH 7.5, with 8.6% sucrose (w/v) in a quartz cuvette, and were illuminated for 15 min with visible light, emitted from a 500‐watt xenon lamp that had passed through two 3‐cm filters of water and a saturated aqueous CuSO_4_ solution and a cutoff filter of 450 nm. Protein was precipitated with ice‐cold EtOH (final concentration 70% (v/v)) at −20 °C for 4 h.

### Chemical fragmentation

Photoaffinity‐labeled plasma membrane proteins (75 µg) were cleaved with BNPS‐skatol [17]. The precipitated protein was dissolved in 7.5 µL of H_2_O and 22.5 µL of the following mixture: 1.3 mg of BNPS‐skatol (Fluka) dissolved in 1 mL of 100% acetic acid. The reaction was carried out at 47 °C for 18 h. The digest was stopped by diluting the mixture with 24 µL of H_2_O. The cleavage products were concentrated in an evaporator and analyzed on a 15 × 17 × 0.1 cm preparative 4%/12% discontinuous Tricine/PAGE [18].

Three milligram plasma membrane protein turned out to be necessary to purify sufficient amounts of samples of peptide for the final Edman sequence analysis. Two gels were used each of which was loaded with 20 photoaffinity‐labeled samples of 75 µg plasma membrane protein.

### Purification of the [^125^‐I]‐Iodomycin‐labeled Skatol fragment

The [^125^I]‐iodomycin‐labeled peptide of lowest molecular weight was gel‐eluted from the discontinuous Tricine gel onto a 3 kDa cutoff membrane in a Centrilutor (Amicon, Merck, Darmstadt, Germany) as described previously [16]. Excess volume was removed by centrifugation of the centricon tubes at 4 °C at 7500 ***g***. This solution was washed three times by ultrafiltration with 3 mL of 5 mm Tris/HCl, pH 7.5, to remove SDS to the greatest possible extent. Subsequently, the concentrated peptide solution was removed from the filtration unit and supplemented with guanidinium hydrochloride to a final concentration of 6 m. The peptide solution was separated on a phenyl column (Macherey & Nagel, ET 50/3 Nucleosil 100‐5 C6H5) by high‐performance liquid chromatography (HPLC) reversed‐phase chromatography as described [16]. Solvent A contains 0.1% trifluoroacetic acid (TFA); solvent B, 60% acetonitrile, 20% isopropanol, and 0.1% TFA.

### Cleavage with Endoproteinase Lys‐C

The HPLC fraction that contained the iodomycin‐labeled peptide was dried in an evaporator and digested with 0.02 µg lysyl endopeptidase from Achromobacter (WAKO 129‐02541, Neuss) at 37 °C for 24 h in 50 mm Tris pH 9; 10% glycerol; 1% SDS; and 0.05% (w/v) bromophenol blue in a maximum volume of 20 µL. Immediately before application to the gel, 0.04 µL mercaptoethanol was added to the sample buffer to act as a scavenger for nonpolymerized acrylamide. The cleavage products were separated on a 13% Tricine/PAGE (const. 40 V, 16 h), and afterward, the gel was exposed to an imaging plate.

### Purification of the Lys‐C cleavage product

The photoaffinity‐labeled peptide was gel‐eluted in a dialysis tube with a molecular weight cutoff of 2 kDa (Serva Spectra/Por 44 166) for 6 h in 50 mm NH_4_HCO_3_ buffer with 0.06% SDS and for a further 15 h in the same buffer without SDS [19]. Without addition of further chemicals, the peptide was purified by HPLC reversed‐phase chromatography (HPLC Basic System 800, 9003‐0800) on a phenyl‐hexyl column (Luna 00B‐4257‐BO; Phenomenex, Aschaffenburg, Germany), which was used together with a security guard (Phenylpropyl AJO‐4350; Phenomenex). The gradient was held at 5% B to inject the sample and run after start in 15 min from 5% to 70% B, in 20 min from 70% to 80% B, in 15 min from 80% to 90% B, in 15 min from 90% to 99% B, 5 min hold at 99% B, and in 5 min from 99% to 5% B. The flow rate was 100 µL·min^−1^. Solvent A contains 0.1% TFA; solvent B, 60% acetonitrile, 20% isopropanol, and 0.1% TFA.

### Analysis of the Lys‐C cleavage product by Edman sequence analysis

Peptides were sequenced on a pulsed liquid‐phase Applied Biosystems 477A protein sequenator with a 120A on‐line HPLC system: The HPLC fractions were acidified with 1% TFA (final) and then applied to ProSorb™ polyvinylidene fluoride sample preparation cartridges. Cartridges were washed and coated with polybrene according to the manufacturer's instructions. The sequenator was run with standard reaction and conversion cycles. All sequencer reagents were obtained from PE Biosystems.

For the on‐line phenylthiohydantoin (PTH) amino acid analysis, we used a Shandon Hypersil ODS Column (250 × 1.6 mm, 5 µm) at 46 °C with buffer A 3.5% tetrahydrofuran (Fluka, spectroscopy grade) adjusted to pH 4 with 9.7 mL of 3 m sodium acetate buffer (pH 3.8) and 0.3 mL triethylamine (Pierce, sequencing grade, Thermo Fisher Scientific, Dreieich, Germany) and buffer B / 100% acetonitrile (Scharlau, ultragradient grade) with 250 nm dimethylphenylthiourea at a flow rate of 125 µL·min^−1^. The gradient was 14 min held at 11% B, in 0.5 min from 11% to 15% B, in 6 min from 15% to 23% B, in 12 min from 23% to 41% B, 9 min held at 41% B, in 0.5 min from 41% to 90% B, and 5 min held at 90% B.

## Results

Digestion of [^125^I]‐iodomycin‐photolabeled Pgp of CHO B30 cells at tryptophan residues with BNPS‐skatol had revealed that iodomycin had been photo‐incorporated into the amino acid sequence fragments 230–312 of hamster Pgp [16]. The aim of this work was to refine the localization of the photobinding site of iodomycin within this Pgp fragment.

### The cleavage of the large about 9 kDa BNPS‐skatol derived iodomycin‐labeled peptide

The contamination of peptides other than the peptide of interest is one major inherent problem of peptide sequencing from proteolytic digests. We minimized this problem by sequential chemical and enzymatic proteolysis at different protein cleavage sites and the purification of first digestion peptides.

**Fig. 1:**
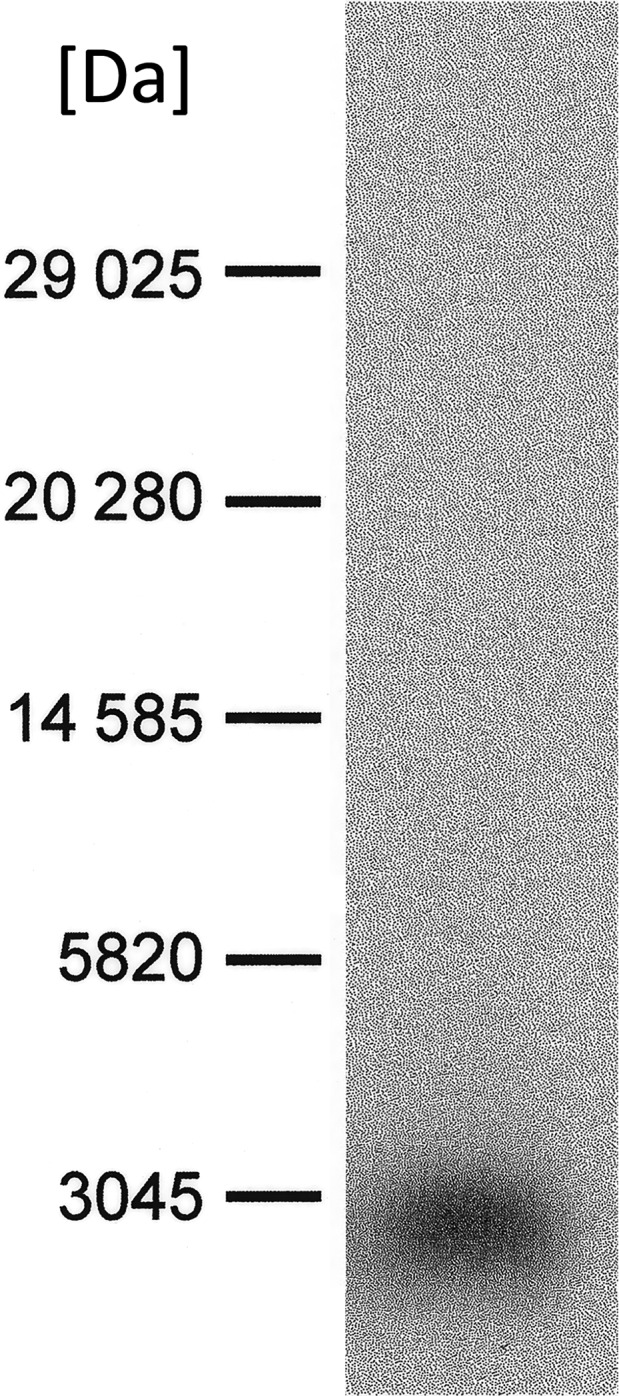
The digest of the iodomycin‐labeled BNPS‐skatol cleavage fragment with endoproteinase Lys‐C. The lane shows the cleavage of a HPLC fraction, which contained the iodomycin‐labeled peptide with the molecular weight of about 9 kDa separated after exposure of the gel to an imaging plate. The radioactive iodomycin‐labeled ‘short’ peptide reproducibly migrated in all 10 experiments within the same molecular weight range below the 3 kDa standard protein.

First, a [^125^I]‐iodomycin‐photolabeled fragment of Pgp was purified from a BNPS‐skatol digestion of a membrane protein preparation. Cleavage with BNPS‐skatol results in an about 9 kDa large iodomycin‐labeled peptide [16]. The purified 9 kDa peptide was then subjected to digestion with endoproteinase Lys‐C, which cleaves directly behind lysine residues. This procedure was necessary because the digestion of the membrane preparation with Lys‐C resulted seldom in the ‘short’ peptide, which was achieved after the cleavage of the 9 kDa large fragment. The protein topology and the insertion of the protein in the membrane may impede the accessibility of the 82 lysines of Pgp so that partial digest becomes possible. The digest of the 9 kDa peptide with endoproteinase Lys‐C in 1% SDS was very effective and always resulted in the same cleavage product (Fig. 1). Only the Achromobacter lysyl endopeptidase turned out to be suitable for efficient cleavage of the 9 kDa peptide. The 9 kDa BNPS‐skatol peptide contains eight lysine residues whereby the nine resulting Lys‐C peptides can be distinguished by different masses. Figure 1 shows the iodomycin‐labeled peptide, which resulted after cleavage with Lys‐C with a mass smaller than the 3 kDa protein standard.

### The purification and identification of the internal ‘short’ Lys‐C‐peptide

**Fig. 2:**
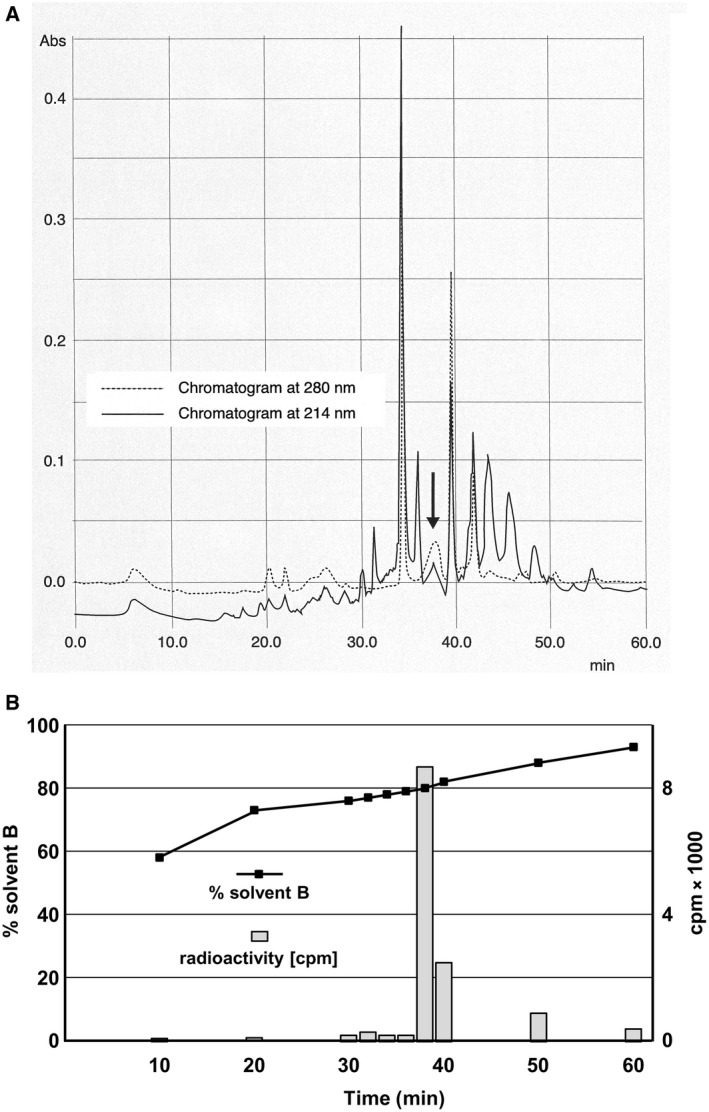
The HPLC of the internal Lys‐C‐photolabeled peptide. (A) Spectrophotometry. The chromatogram shows the separation of the gel‐eluted iodomycin‐labeled ‘short’ peptide on a special hydrophilic‐coated phenyl column (Luna; Phenomenex). The arrow indicates the fraction, which contained the radioactivity. This fraction was applied to a ProSorb™ cartridge and sequenced. The peak was rather broad and absorbed more strongly at 280 nm than at 214 nm indicating that this fraction carried the covalently linked product of the photoreaction with iodomycin consistent with our knowledge that during illumination of iodomycin the broad asymmetric absorption band of the anthraquinone residue between 350 and 580 nm disappears and a symmetric absorption band between 260 and 350 nm emerges [15]. (The extinction data were achieved by using a diode array detector (TIDAS 256) and the software program Spectrachrom both delivered from the Company J & M in Aalen.) (B) Solvent gradient and distribution of [^125^I]‐iodomycin. The fraction that we later on used for sequencing (indicated by an arrow in A) eluted always at a high concentration of solvent B (from 78% to 84% B seen during independent experiments) and contained the maximal amount of radioactivity as indicator for the iodomycin‐labeled peptide.

As the first purification step, the radioactive peptide cut off the gel guided by the phosphorimager signal. After electroelution, again HPLC was necessary to remove co‐migrating peptides. A phenyl column with a special hydrophilic coating was the only matrix, which showed reasonable results with regard to separation and recovery. Figure 2A shows the chromatogram. For sequence analysis, we used the HPLC fraction with maximal radioactivity (Fig. 2B).

**Fig. 3:**
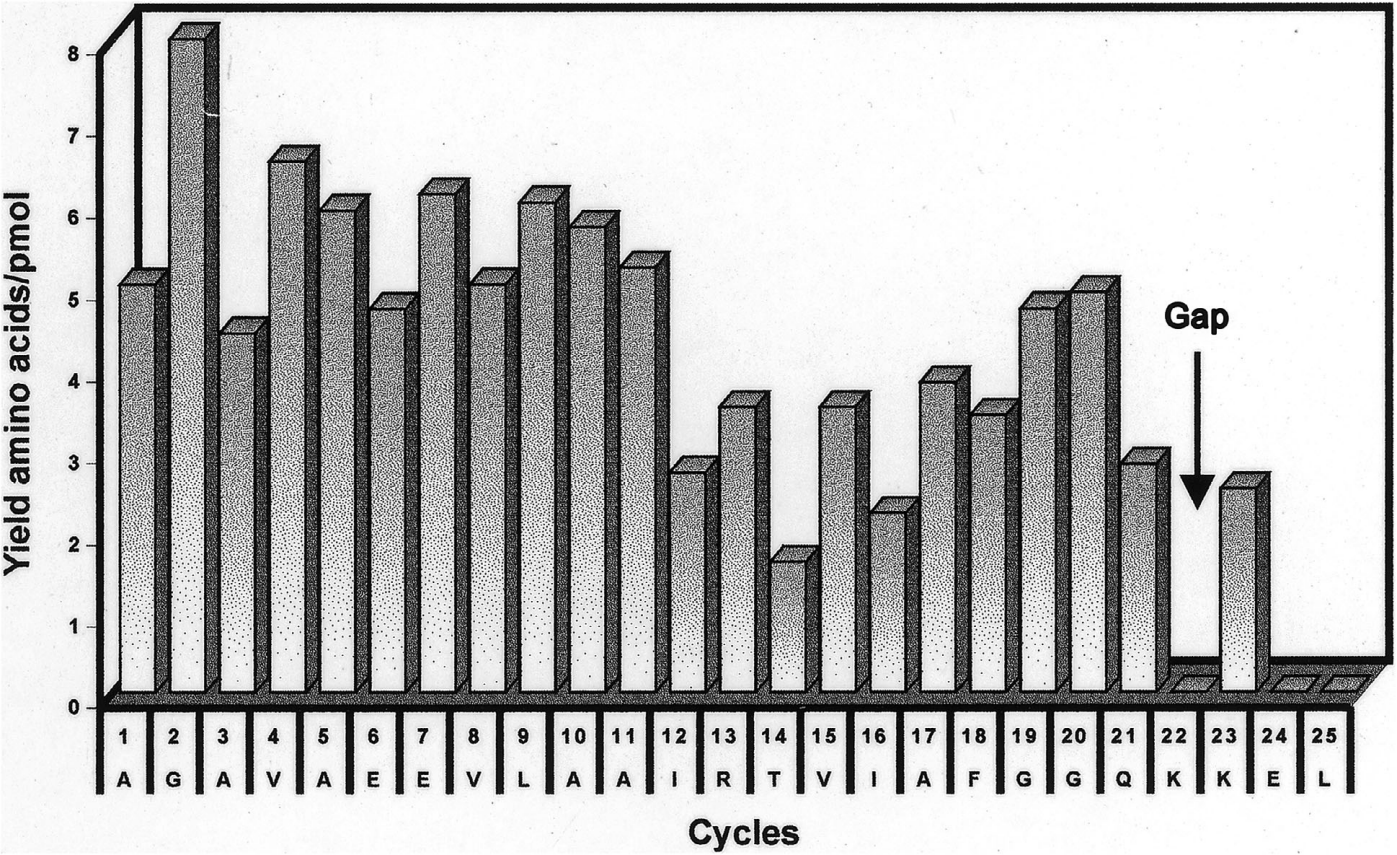
Yields of PTH amino acids from the iodomycin‐cross‐linked peptide obtained by Lys‐C treatment. Axis of ordinates: Yield of PTH amino acids in pmol.

The Edman sequence analysis revealed the high purity of this fraction. Only one peptide could be read throughout the reaction cycles (Fig. 3). The sequence we found was part of the iodomycin‐labeled 83 amino acid large fragment identified in our previous work [16].

### Identification of the iodomycin‐binding site: The Edman sequence analysis as the tool to identify the iodomycin‐labeled amino acid

The sequencing run proceeded for 25 cycles with yields in the picomolar range. A sequence of 23 amino acids stretching from amino acid residue 247 to position 269 in the hamster Pgp primary sequence could be read unambiguously with the remarkable exception of the amino acid at position 22. At this position, a gap was noticed instead of the expected lysine (lysine no. 20 of the 82 lysine residues located at position 268 in the Pgp primary sequence; Fig. 3 ). However, the following amino acid (again a Lys residue) was sequenceable within the expected yield range and its side chain was recognized by the lysyl endopeptidase because at this position the peptide stops (as expected from the cleavage specificity of Lys‐C). The next amino acids of the corresponding Pgp sequence Glu and Leu did not show up. However, there was a slightly higher‐than‐expected lag of lysine in amino acid 24 (0.9 pmol), indicating that the modification at Lys no. 20 to a certain degree renders the cleavage at the following lysine more difficult. We analyzed the filter after the sequencer run for any remaining radioactivity on the filter. The amount was reduced to background confirming that the [^125^I]‐iodomycin‐cross‐linked amino acid had been identified.

**Fig. 4:**
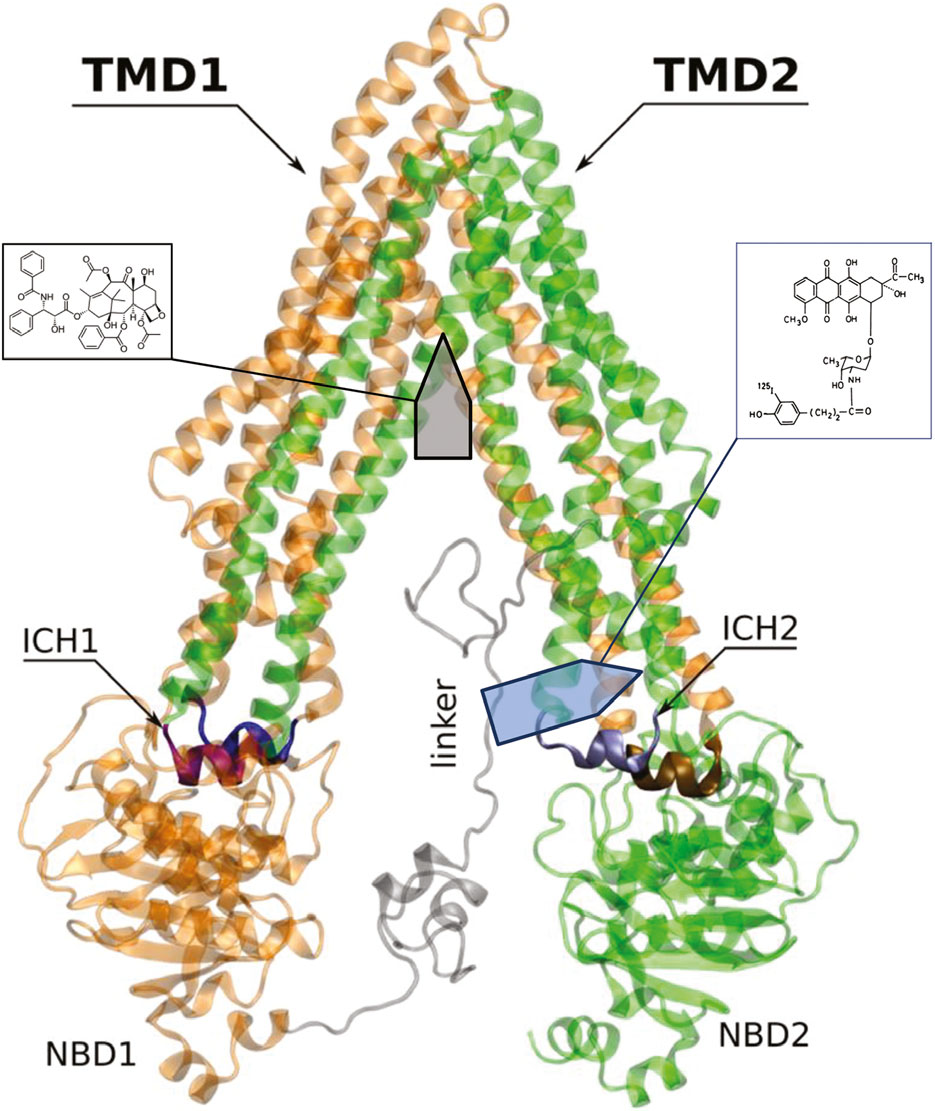
Localization of the binding sites of taxol [8] (gray arrow) and iodomycin (blue arrow, this work) in the IF of human Pgp [33]. The two TMDs (TMD1: orange and TMD2: green) are linked to the NBDs NBD1 and NBD2 by coils and intracellular coupling helices (ICH1 (purple)/ICH4 (blue) with NBD1 and ICH2 (silver)/ICH3 (brown) with NBD2). The iodomycin‐photolabeled amino acid Lys 271 resides within the first turn of transmembrane helix TMH5 adjacent to ICH2. The drug‐binding pocket for taxol was resolved by Cryo‐EM [8]. The structural representation of the human Pgp in an IF is republished from the article by Bonito *et al*. [33]. This open‐access article is licensed under a Creative Commons Attribution 4.0 International License (http://creativecommons.org/licenses/by/4.0/). Arrows and the structural formulas of taxol and iodomycin were inserted into the original figure.

## Discussion

Photoaffinity labeling of plasma membrane vesicles of MDR CHO B30 cells with [^125^I]‐iodomycin, subsequent sequential cleavage with BNPS‐skatol and endoproteinase Lys‐C, and the Edman sequencing of the purified photoaffinity‐labeled peptide identified the lysine residue at position 268 in the Pgp primary sequence as the major photobinding site of iodomycin in CHO B30 cells.

Of the large number of structurally diverse compounds that are known to interact with Pgp, iodomycin has unique chemical features that allow the study of the poly‐specific binding site of Pgp with several complementary approaches. Like any other substrate of Pgp, exposure of cell lines to iodomycin will induce the overexpression of Pgp and the concomitant phenotype of cross‐resistance and collateral sensitivity to a broad range of xenobiotics [14, 15]. However, in addition the thermodynamics and kinetics of drug binding to Pgp can be investigated in its native environment of the plasma membrane by monitoring changes in fluorescence of the anthracycline residue of iodomycin [15]. According to spectrofluorometric titrations, iodomycin like its parent compound daunomycin binds to Pgp with an affinity constant of about 5 × 10^7^ m ^−1^. The association with its binding sites in the plasma membrane is a diffusion‐controlled process with a rate constant of more than 10^9^ m ^−1^·s^−1^. Thus, the selectivity of drug binding is reflected in the dissociation rate, which is thousand‐fold or more faster than the covalent photolabeling of Pgp with iodomycin [14, 15]. Hence, we conclude that radioiodinated iodomycin will photolabel its major equilibrium binding site on Pgp that is accessible by diffusion from the solution. Moreover, due to the high activity of [^125^I]‐iodomycin (2000 Ci·mmol^−1^) a low amount of 0.1 pmol iodomycin per 10 µg protein could be chosen to achieve specific photo‐incorporation of iodomycin into Pgp and not any other plasma membrane protein.

Despite these many advantages of iodomycin for mechanistic studies on Pgp, some limitations of our experimental approach need to be mentioned, that is, the photobinding site identified here *in vitro* does not necessarily match with all Pgp iodomycin‐binding sites during the substrate‐coupled conformational cycle of Pgp *in vivo*. Pgp‐containing plasma membrane vesicles were prepared from CHO B30 cells by ultrasonication and several ultracentrifugation steps. Storage and photolabeling were performed in standard buffered sucrose solution without ATP. The native topology of the membrane in the cell and the distribution of Pgp conformations may have been affected by this procedure. Hence, photolabeling may have covalently linked iodomycin to a subpopulation of Pgp conformational states whose drug‐binding sites were accessible under our *in vitro* conditions. Since we examined photobinding in the absence of ATP, most hamster Pgp was probably in the IF conformation with a large binding cavity and open portals for drug entry related to the apo murine Pgp structure resolved by X‐ray crystallography [3]. On the other hand, combining the data gained from our thermodynamic, kinetic, and photoaffinity labeling studies the evidence is strong that our analytical approach of cleavage and sequencing to search for the amino acid that photo‐incorporated iodomycin identified a major drug‐binding site of hamster Pgp.

In our previous studies, the iodomycin photo‐cross‐linking position had been narrowed down to amino acids 230–312 in the primary sequence of CHO Pgp [16]. The Edman sequencing of this fragment identified no cross‐linked amino acids in the 17 N‐terminal amino acids. Within the frame of this study, we had to develop an additional proteolysis protocol, which takes care of the very hydrophobic nature of the peptide–iodomycin cross‐link. The only protease useful for that approach was lysyl endopeptidase from *Achromobacter lyticus,* which cleaved specific even at high SDS concentrations. The photolabeled peptide thereby remained in a detergent‐containing solution until the last HPLC purification step, which enabled the recovery of sequenceable amounts of cross‐linked ≈ 3 kDa peptide after purification. The whole peptide could be sequenced matching to 100% with the hamster Pgp sequence except that the second but last amino acid, that is, lysine 268 did not show up, suggesting that this amino acid was cross‐linked with iodomycin. A gap in a sequence is typical for modified amino acids. Often such a covalent modification, in this case the linkage with the affinity label, leads to a drop in sequencing yield after the cross‐link position, but in our case the Edman sequencing cycle was only moderately impaired so that the second non‐cross‐linked lysine 269 showed up with reasonable yield.

Iodomycin photolabeled lysine 268 in hamster Pgp that is homologous to lysine 271 in human Pgp. The amino acid sequence of the 3 kb photolabeled peptide is 100% conserved in the hamster, murine, and human orthologs [3]. Lysine 271 of human ABCB1 resides adjacent to the terminal amino acid of TMH5 on the intracellular side in vicinity to the NBDs (Fig. 4) [3, 5, 6, 8]. According to our thermodynamic and kinetic analyses (see above), this location should present the equilibrium binding site of ATP‐free Pgp for daunomycin and iodomycin in plasma membranes of MDR CHO B30 cells. Since our plasma membranes were not supplemented with ATP, Pgp should reside in the IF conformation. The photolinked lysine 271 is thus located in the region with the maximal intramolecular distance between the two membrane‐spanning domains in the absence of ATP, but in the region with low distance in the high‐energy post‐ATP hydrolysis state [3, 4, 6, 8]. The distance between aa276 in TMD5 and aa360 in TMD6 is between 4 and 5 nm in the IF nucleotide‐free conformation but 2 nm or less in the OF conformation and the post‐ATP hydrolysis state [6].

In the surroundings of Lys271, the binding cavity should be large and flexible in the absence of ATP and smoothly accommodate rigid and bulky ligands such as iodomycin. A large cavity close to the cytosolic NBDs could also explain why the uptake of iodomycin into the plasma membrane is a diffusion control process with a rate constant of more than 10^9^ m ^−1^·s^−1^. We moreover know from fluorescence spectroscopy of the plasma membrane of drug‐sensitive CHO AuxB1 and MDR B30 cells with *n*‐(9‐anthroyloxy) fatty acids (*n* = 2, 7, 9, 12, 16) that the fluidity gradient along the transverse plane of the plasma membrane is smoother in MDR cells [20]. Compared to drug‐sensitive AuxB1, the rotational mobility is twofold higher in the inner third of the bilayer leaflet of B30, which will facilitate the diffusion of ligands to the binding cavity of Pgp in MDR cells.

In total, 33 amino acid residues were identified in mouse apo PgP by X‐ray crystallography that were close to the ligands QZ59‐RRR or QSZ59‐SSS in TMHs 1, 5, 6, 7, 8, 9, 11, and 12 or with verapamil in TMHs 1, 6, 7, 10, and 12 [3]. With the exception of TMH6 and TMH12, the binding subsites reside in the center or the ectoplasmic site of the bilayer leaflet. Similarly, human ABCB1 reconstituted in nanodiscs was examined in its complexes with taxol (Fig. 4) or zosuquidar by cryo‐EM [8]. Thirty‐two amino acid residues of TMHs 1, 4, 5, 6, 7, 8, 9, 10, 11, and 12 made contact with the ligands, all of which with the exception of TMH6 localized in the center or the ectoplasmic site of the bilayer. In other words, as shown in Fig. 4 the binding pockets identified by X‐ray [3] or cryo‐EM [8] do not match with the equilibrium binding site at the border between TMDs and NBDs reported here for Pgp in MDR B30 membranes. The different chemical structure of the ligands may be one reason for the nonoverlapping location of the binding sites. However, we consider it more likely that different conformations of Pgp are selected during the isolation of native plasma membranes from MDR cells and during the reconstitution of recombinant protein in a model membrane. Rate constants of (3–5) × 10^9^ m ^−1^·s^−1^ seen for the association of daunomycin and iodomycin with Pgp [15] are highly unlikely to occur for the entry of ligand into the well‐defined binding pockets resolved by the X‐ray [3, 5] and cryo‐EM [8] studies (Fig. 4). The different experimental approaches apparently scanned contact residues for ligand binding in Pgp that differ in affinity and kinetics from each other consistent with the tasks of Pgp to change its conformation during the cycle of ligand recognition, transport, and delivery to the extracellular milieu. The numerous studies on photo‐incorporation [21, 22, 23, 24, 25] or transport kinetics [26] of ligands and on site‐directed mutagenesis scanning [27, 28, 29, 30, 31] have demonstrated that Pgp possesses spatially distinct and overlapping binding sites to accommodate chemically diverse compounds [32].

## Conflict of interest

The authors declare no conflict of interest.

## Author contributions

AD and BT conceived the study. AD, HT, and MR performed experiments and analyzed the data and BT wrote the manuscript.

## Data Availability

Data will be available from the corresponding author upon reasonable request.
